# Two‐Sample Mendelian Randomization Analyses Identified Lipid Species Associated With Intracranial Aneurysm Formation

**DOI:** 10.1002/brb3.70435

**Published:** 2025-03-18

**Authors:** Junqing Yan

**Affiliations:** ^1^ Nanxiang Branch of Ruijin Hospital Shanghai China

**Keywords:** intracranial aneurysm, lipids, Mendelian randomization, single nucleotide polymorphisms

## Abstract

**Objectives:**

Intracranial aneurysm (IA) poses a significant health risk, and its formation involves various factors, including lipid metabolism, while former research only focused on the standard lipid. The purpose of this study is to explore 179 lipid variants' impact on unruptured intracranial aneurysms (uIA).

**Materials and Methods:**

Utilizing GWAS data for lipids and uIAs, MR analyses were employed with pleiotropy, heterogeneity, and sensitivity tests. Reverse MR analyses were then conducted.

**Results:**

MR analyses revealed seven lipids associated with uIAs: TAG (51:3). SE (27:1/16:1), PC (18:2_18:2), TAG (48:1), TAG (48:2), and TAG (51:3) were identified as uIA risk factors, while SE (27:1/18:1) and SM (d34:0) exhibited protective effects. Reverse MR analysis showed no bidirectional causal relationships.

**Conclusions:**

This study identifies specific lipid variants causally linked to uIAs, shedding light on their roles in IA formation. These findings contribute to future research on IA risk assessment and potential therapeutic interventions.

## Graphical abstract

This study investigates the causal relationship between lipid variants and unruptured intracranial aneurysm (uIA) formation using Mendelian randomization. Seven lipid species were identified as risk factors or protective agents for uIAs, offering insights into potential new biomarkers for IA risk assessment and treatment.

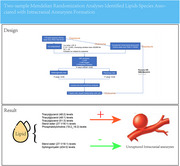

## 1 Introduction

Intracranial aneurysm (IA) is a type of intracranial vascular disorder, and different epidemiological studies show that the incidence rate of IA varies between 2% and 7% in different populations (Freneau et al. 2022; Vlak et al. 2011; Brown and Broderick 2014). Most unruptured intracranial aneurysms (uIAs) are incidentally detected through neuroimaging examinations, typically without specific symptoms, although a few may present focal neurologic dysfunction due to a positional effect (Cianfoni et al. 2013; Goldenberg‐Cohen et al. 2004). However, although the risk of rupture is low, the most dangerous and common manifestation is an aneurysmal subarachnoid hemorrhage (SAH), with a high mortality rate of up to 50% and a complication rate of 70% once it occurs (J. Jin et al. 2022). Therefore, the prevention and treatment of IA formation are crucial topics in the realm of intracranial vascular diseases.

The formation of IAs is multifactorial, involving factors such as variations in parent arterial anatomy, abnormal hemodynamic flow, oxidative stress, and inflammatory reactions (Bor et al. 2008; Etminan and Rinkel 2016; Cebral et al. 2017; Hackenberg et al. 2020). Previous studies have identified several risk factors associated with the development of cerebral aneurysms, including smoking, female gender, positive family history, alcohol consumption, hypertension, advanced age, and certain genetic conditions (Vlak et al. 2011; Lindgren et al. 2014). In addition to these factors, researchers have turned their attention to the role of lipid metabolism in IAs recently (Frosen et al. 2013; Lovik et al. 2021; Ou et al. 2020). Disruptions in lipid metabolism can impact the progression of IAs through various mechanisms, including inducing systemic inflammation and oxidative stress, altering the lipid composition and metabolism of the intracranial arterial wall, weakening the structural strength and elasticity of the intracranial arterial wall, and regulating the expression and activity of various genes, proteins, and related signaling pathways (Vanrossomme et al. 2015).

Past research has traditionally measured plasma lipids using standard lipid profiling, including high‐density lipoprotein cholesterol (HDL‐C), low‐density lipoprotein cholesterol (LDL‐C), triglycerides (TG), and total cholesterol (TC). However, a recent genome‐wide association study (GWAS) has significantly expanded our understanding of circulating lipid variability and diversity using modern high‐throughput lipidomics technologies. This study explored 179 lipid variants across 13 major classes, including cholesterol esters (CE), ceramides (CER), cholesterol (Chol), diacylglycerols (DAG), lysophosphatidylcholines (LPC), lysophosphati‐dylethanolamine (LPE), phosphatidylcholines (PC), phosphatidylcholine ethers (PCO), phosphatidylethanolamines (PE), phosphatidylethanolamine ethers (PEO), phosphatidylinositol (PI), sphingomyelins (SM), and triacylglycerols (TAG). In comparison to standard lipids, this research has improved cardiovascular disease (CVD) risk assessment and provided new therapeutic options for CVDs (Ottensmann et al. 2023).

Given the impact of lipids on IAs, as mentioned earlier, and the similarity between many risk factors for IAs and CVDs, we hypothesize that exploring these lipid variants may also contribute to the prevention and treatment of IAs. Therefore, we conducted a Mendelian randomization (MR) study to investigate whether there is a causal relationship between these 179 lipids and the formation of IAs. Due to our study's focus on the formation of IAs, we exclusively considered data related to uIAs and did not include data on SAH.

MR analysis is a method that establishes causal inference by leveraging naturally occurring genetic variations in the population. It uses genetic variants as instrumental variables (IVs), effectively addressing issues related to confounding and reverse causation (Hong et al. 2023). This approach helps clarify relationships between variables.

## 2 Method

### 2.1 Study Design

**FIGURE 1:**
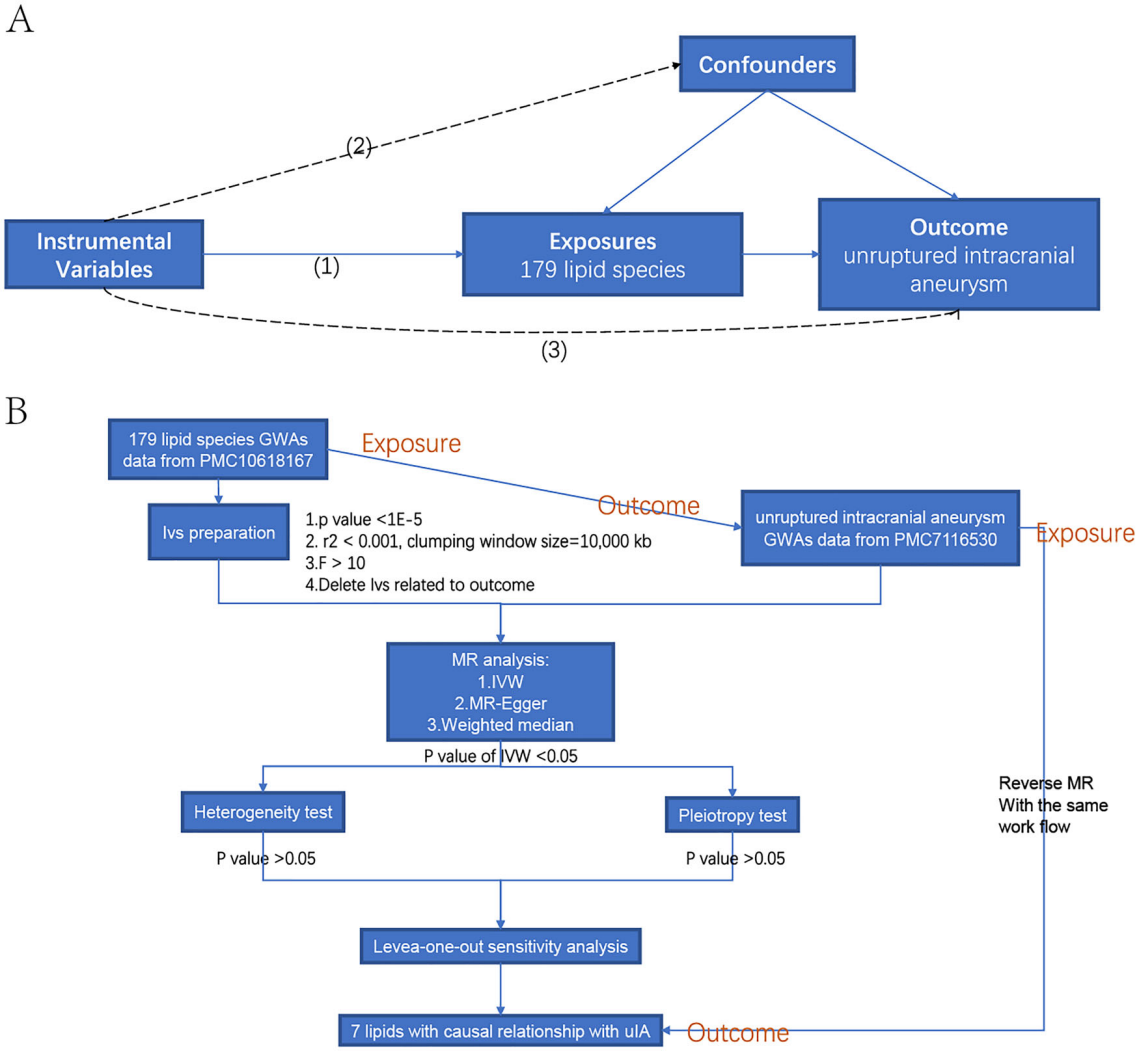
(A) Basic principles of Mendelian randomization. (B)Work flow of the present study.

Figure 1A demonstrates the basic principle of MR analysis. Three conditions are required for MR analysis: (1) robust connection between IVs and exposure factors (Sekula et al. 2016); (2) absence of correlation between IVs and confounders (Jia et al. 2023); (3) IVs exclusively influence outcomes through exposure factors, excluding any involvement of alternative pathways (Q. Jin et al. 2023). The analysis flow is displayed in Figure 1B.

### 2.2 Data Source and Preparation

We obtained GWAS data for 179 lipid species from PMC10618167, derived from 7174 Finnish individuals, categorized into 13 classes of lipid (Ottensmann et al. 2023). The GWAS data for UIAs were conducted by The International Stroke Genetics Consortium (ISGC) intracranial aneurysm working group (PMC7116530) (Bakker et al. 2020). The dataset comprises 2070 controls and 71,952 cases.

The IVs were selected by the following standard: Single nucleotide polymorphisms (SNPs) associated with each lipid species at a locus‐wide significance threshold (*p *< 1e−5) were chosen as potential IVs (Zeng et al. 2023). A linkage disequilibrium (LD) window analysis was conducted for all IVs then (*R* ^2^ < 0.001, clumping window size = 10,000 kb). To ensure SNPs are strongly correlated with exposure factors, only SNPs with an *F* value greater than 10 are retained. The formula for calculating the *F* value is as follows (Pierce et al. 2011; Shim et al. 2015):

$$F=\left(N\;-\;2\right)\times R^{2}/\left(1\;-\;R^{2}\right)$$

$$R^{2}=\left(2\;\times \beta^{2}\right)/\left(2\;\times \beta^{2}+2\times N\;\times {\;\mathrm{SE}}^{2}\right)$$

At last, SNP associations with any confounding factors potentially linked to the outcome were excluded using the Phenoscanner website (http://www.phenoscanner.medschl.cam.ac.uk/).

### 2.3 MR Analysis

We conducted MR analysis using the inverse‐variance weighting (IVW), weighted median (WM), and MR‐Egger methods. The primary results are based on the IVW method, while the WM and MR‐Egger methods serve as supplementary analyses and offer broader confidence intervals. It is important to note that the IVW method can only be applied after the impact of statistics influenced by horizontal pleiotropy is mitigated. To address this, we performed a test for horizontal pleiotropy (Burgess et al. 2016). Furthermore, we excluded results with heterogeneity among IVs during Cochran's *Q* test (*p* > 0.05) (Slob and Burgess 2020). Finally, we performed a leave‐one‐out sensitivity analysis for lipids with a *p* value of the IVW method < 0.05 of statistically significant causal relationships to arrive at our final results.

To bolster result credibility, we applied the same MR analysis methods described earlier to conduct a reverse MR analysis using uIAs as the exposure and lipids causally associated with uIAs as the outcomes.

All the mentioned analyses were carried out by the R package “TwoSampleMR” (Hemani et al. 2017, Hemani et al. 2018).

## 3 Results

**FIGURE 2:**
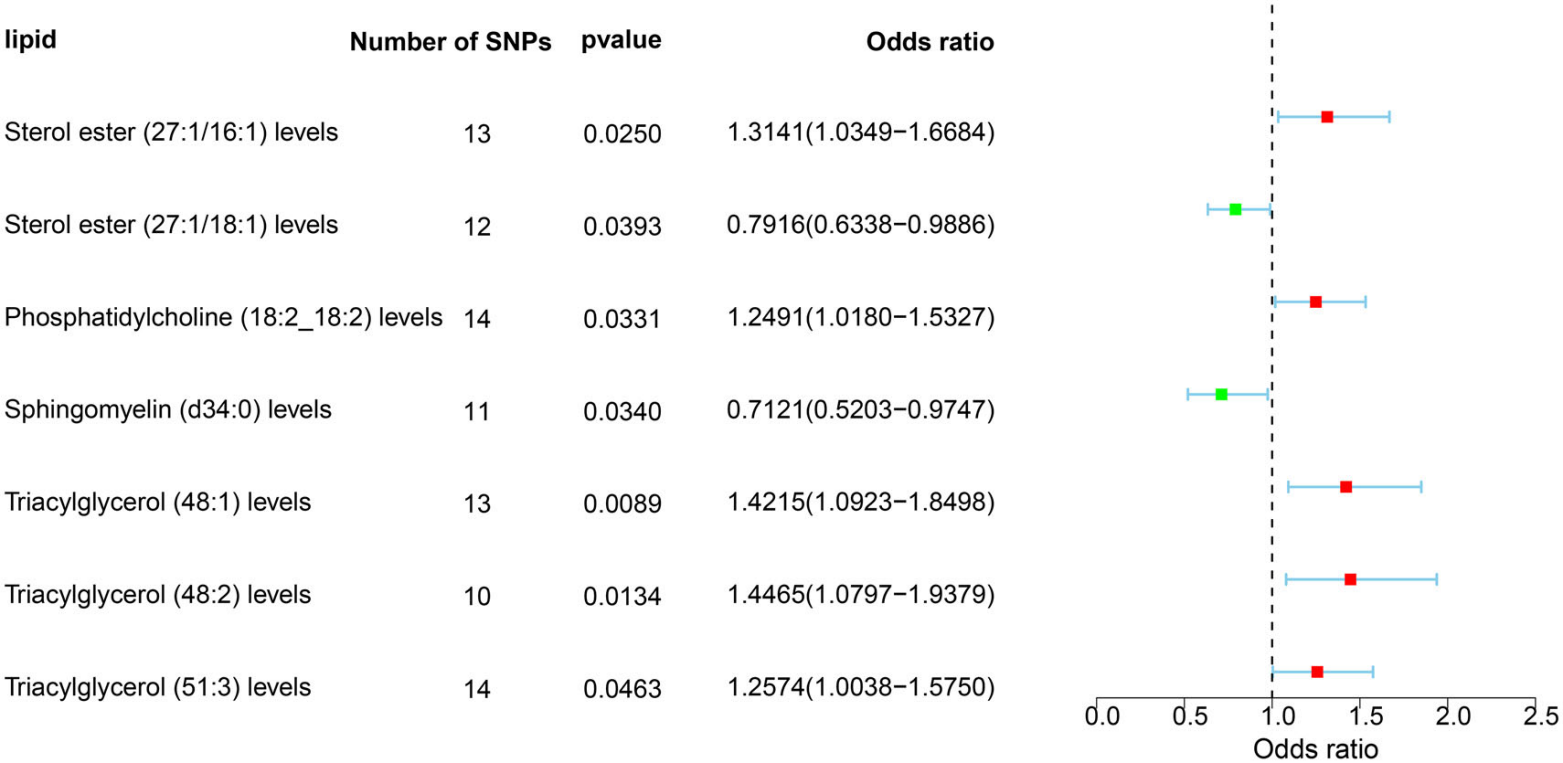
Forest plot of lipids associated with uIA (*p* < 0.05) identified by IVW method.

Following the outlined steps, we conducted MR analysis with various lipid types as exposure factors and uIA as the outcome. Through horizontal pleiotropy tests and heterogeneity tests, we excluded certain statistical data affected by heterogeneity or horizontal pleiotropy, selecting results with *p* values exceeding 0.05 in both the heterogeneity *Q*‐test and the pleiotropy test. As shown in Figure 2, the IVW analysis revealed associations between uIAs and seven lipids (SE [27:1/16:1], SE [27:1/18:1], PC [18:2_18:2], SM [d34:0], TAG [48:1], TAG [48:2], TAG [51:3]). However, none of these lipids demonstrated a causal relationship with uIAs in MR‐Egger and WM analyses.

**FIGURE 3:**
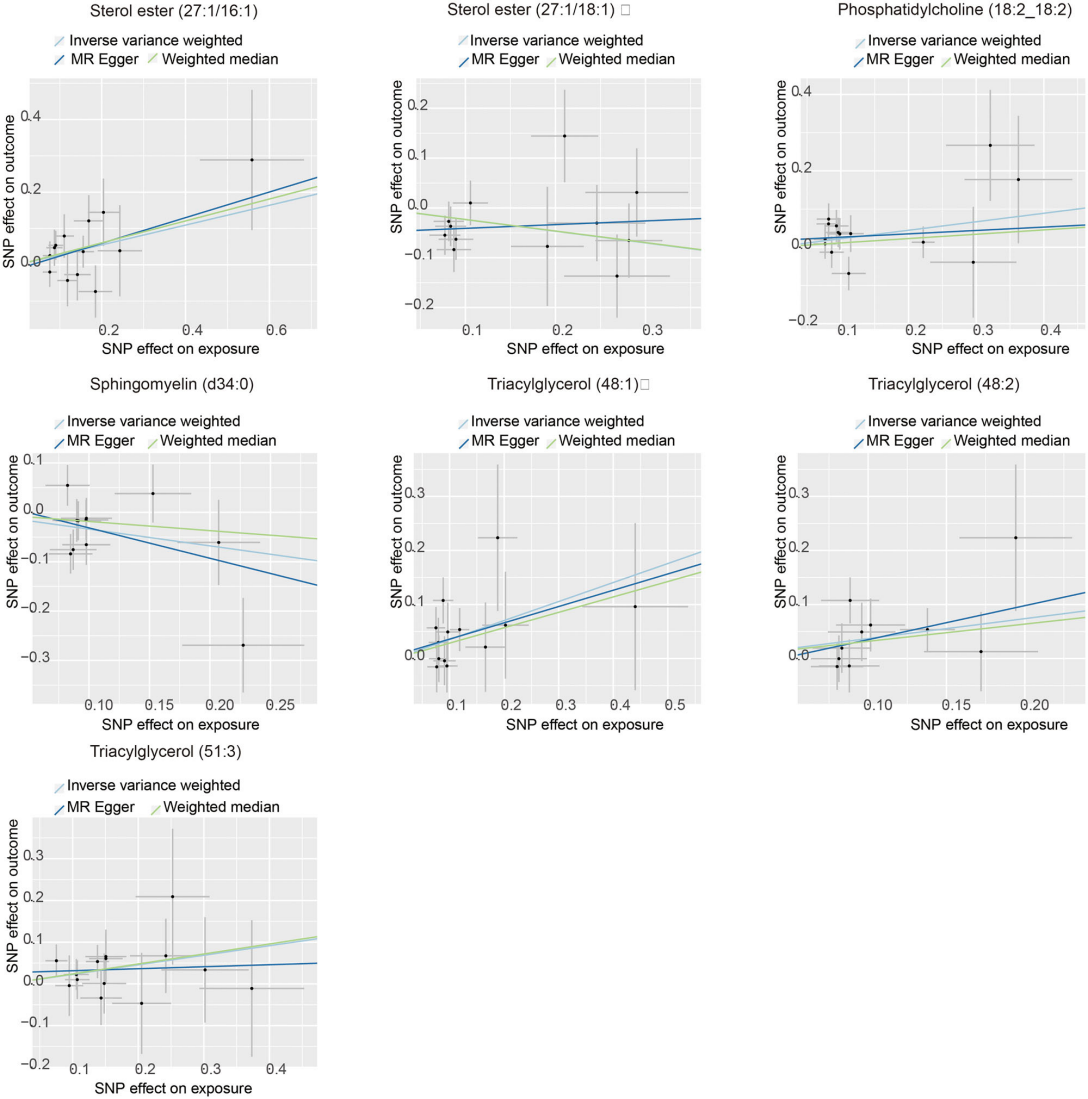
Scatter plots for the causal association between lipids and uIA identified by IVW method.

**FIGURE 4:**
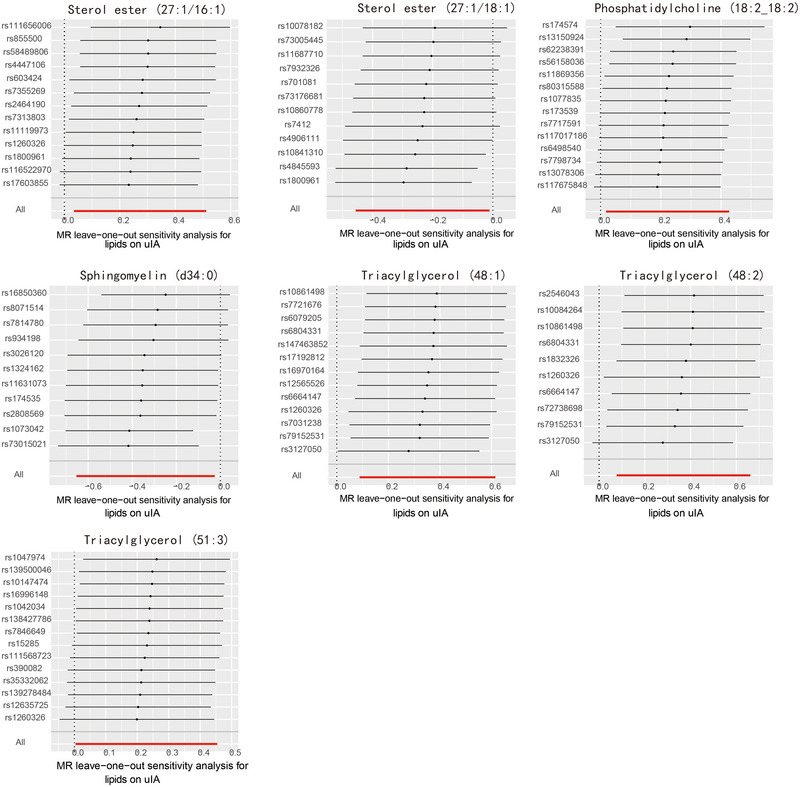
Leave‐one‐out plots for the causal association between lipids and uIA identified by IVW method.

Figure 2 illustrates that the causal relationships of these seven lipids, as per IVW results, persist after sensitivity analyses. Detailed information on the SNPs used in IVW and the results of the MR analysis can be found in Tables 1 and 2. IVW results demonstrated in Figure 3 indicate that SE (27:1/16:1) (IVW, *p* = 0.025, OR = 1.314, 95% CI = 1.035–1.668), PC (18:2_18:2) (IVW, *p* = 0.033, OR = 1.249, 95% CI = 1.018–1.533), TAG (48:1) (IVW, *p* = 0.009, OR = 1.421, 95% CI = 1.092–1.850), TAG (48:2) (IVW, *p* = 0.013, OR = 1.446, 95% CI = 1.080–1.938), and TAG (51:3) (IVW, *p* = 0.046, OR = 1.257, 95% CI = 1.004–1.575) are risk factors for uIAs. Contrarily, SE (27:1/18:1) (IVW, *p* = 0.039, OR = 0.791, 95% CI = 0.634–0.989) and SM (d34:0) (IVW, *p* = 0.034, OR = 0.712, 95% CI = 0.520–0.975) exhibit a protective effect against uIAs. The leave‐one‐out sensitivity analysis results in Figure 4 confirm the sensitivity of these results.

**TABLE 1:** MR results of lipids and uIA with statistical significance by IVW methods.

Lipid	Method	nsnp	*β*	SE	*p* value	lo_ci	up_ci	OR	OR_lci95	OR_uci95	*p* value of Cochran's *Q* test	*p* value of plieotropy test
Sterol ester (27:1/16:1)	Inverse variance weighted	13	0.27311567	0.12182052	0.02496452	0.03434745	0.51188388	1.31405223	1.03494414	1.66843136	0.61905496	0.762075658
	MR‐Egger	13	0.35302978	0.28483886	0.24098861	−0.2052544	0.91131395	1.42337353	0.81444011	2.48758896	0.542262508	
	Weighted median	13	0.30191559	0.1656036	0.06828489	−0.0226675	0.62649864	1.35244706	0.97758752	1.87104788		
Sterol ester (27:1/18:1)	Inverse variance weighted	12	−0.2337532	0.11339833	0.03927007	−0.4560139	−0.0114924	0.79155718	0.63380504	0.98857336	0.4194889	0.146435665
	MR‐Egger	12	0.07451407	0.22552471	0.74791335	−0.3675144	0.51654249	1.0773605	0.69245339	1.67622207	0.550376559	
	Weighted median	12	−0.2318063	0.16129725	0.15067897	−0.5479489	0.08433631	0.79309972	0.57813439	1.08799473		
Phosphatidylcholine (18:2_18:2)	Inverse variance weighted	14	0.22245343	0.10437085	0.03305825	0.01788656	0.42702029	1.24913764	1.01804749	1.53268376	0.47181124	0.565471847
	MR‐Egger	14	0.08830073	0.25043574	0.73051238	−0.4025533	0.57915478	1.09231657	0.66861069	1.78452948	0.419259942	
	Weighted median	14	0.11346516	0.14452744	0.43240845	−0.1698086	0.39673893	1.12015286	0.8438263	1.48696768		
Sphingomyelin (d34:0)	Inverse variance weighted	11	−0.3395189	0.16014051	0.03399497	−0.6533943	−0.0256435	0.71211283	0.52027679	0.97468249	0.136447755	0.635896397
	MR‐Egger	11	−0.6151872	0.58680091	0.32180243	−1.765317	0.53494259	0.5405397	0.17113253	1.70735023	0.10578214	
	Weighted median	11	−0.1852536	0.18719284	0.32235003	−0.5521515	0.18164442	0.83089358	0.57570982	1.19918771		
Triacylglycerol (48:1)	Inverse variance weighted	13	0.35168041	0.1343958	0.00887709	0.08826465	0.61509617	1.42145417	1.09227716	1.84983449	0.741592836	0.877826065
	MR‐Egger	13	0.30160055	0.34549716	0.40134661	−0.3755739	0.97877498	1.35202106	0.68689497	2.66119424	0.666491067	
	Weighted median	13	0.28499869	0.17973416	0.11281466	−0.0672803	0.63727764	1.32976028	0.93493314	1.89132499		
Triacylglycerol (48:2)	Inverse variance weighted	10	0.36914213	0.14922402	0.01337052	0.07666306	0.66162121	1.44649318	1.07967822	1.93793158	0.568789918	0.651940704
	MR‐Egger	10	0.62913727	0.57468717	0.30548918	−0.4972496	1.75552411	1.8759914	0.60820117	5.78647971	0.489983389	
	Weighted median	10	0.31876718	0.20786652	0.12514813	−0.0886512	0.72618556	1.37543106	0.91516472	2.06718042		
Triacylglycerol (51:3)	Inverse variance weighted	14	0.22901092	0.1149198	0.04628454	0.00376812	0.45425372	1.25735577	1.00377523	1.57499756	0.97287093	0.554358558
	MR‐Egger	14	0.04803179	0.31896135	0.88280231	−0.5771325	0.67319603	1.04920401	0.5615062	1.96049312	0.966326309	
	Weighted median	14	0.23930311	0.14981931	0.11020371	−0.0543427	0.53294895	1.27036353	0.94710745	1.70394976		

**TABLE 2:** SNPs used in MR analyses.

Lipid	SNP	*β*	SE	Sample size	*p* value	*R* ^2^	*F*
Sterol ester (27:1/16:1)	rs11119973	0.110738	0.0231985	7172	1.84338e−06	0.00316706	22.7799317
	rs111656006	0.185117	0.0400311	7172	3.81832e−06	0.00297279	21.3784608
	rs116522970	0.55842	0.12419	7172	7.01117e−06	0.00281116	20.2128498
	rs1260326	−0.0904035	0.0174668	7172	2.32931e−07	0.00372121	26.7807459
	rs17603855	0.169155	0.0356528	7172	2.1289e−06	0.00312882	22.5040803
	rs1800961	−0.204194	0.0372477	7172	4.34267e−08	0.00417283	30.044564
	rs2464190	0.0764228	0.0167672	7172	5.24998e−06	0.00288821	20.7684304
	rs4447106	0.118474	0.0242138	7172	1.01459e−06	0.00332685	23.9331067
	rs58489806	−0.142087	0.0315082	7172	6.59485e−06	0.00282743	20.3301404
	rs603424	−0.155845	0.0259739	7172	2.06545e−09	0.00499455	35.9907
	rs7313803	0.0873092	0.0194117	7172	6.96926e−06	0.00281274	20.2242186
	rs7355269	0.243229	0.0529062	7172	4.35121e−06	0.00293832	21.1298515
	rs855500	0.0761958	0.0168529	7172	6.24211e−06	0.00284208	20.4358005
Sterol ester (27:1/18:1)	rs10078182	0.268326	0.0586987	7174	4.92541e−06	0.00290432	20.8904337
	rs10841310	−0.105591	0.019433	7174	5.69845e−08	0.00409854	29.5156949
	rs10860778	−0.0815118	0.0167481	7174	1.15667e−06	0.00329092	23.6803864
	rs11687710	0.08949	0.0191687	7174	3.08593e−06	0.0030289	21.7892661
	rs1800961	−0.21016	0.037228	7174	1.71172e−08	0.00442257	31.8595486
	rs4845593	−0.290262	0.0571029	7174	3.80059e−07	0.00358873	25.8311027
	rs4906111	−0.245889	0.0540683	7174	5.50919e−06	0.00287462	20.6762455
	rs701081	−0.0836375	0.0171434	7174	1.08975e−06	0.0033068	23.795075
	rs73005445	−0.087242	0.0191832	7174	5.50635e−06	0.00287473	20.6770237
	rs73176681	0.191002	0.0404108	7174	2.32683e−06	0.00310434	22.333657
	rs7412	−0.281493	0.0371564	7174	4.00681e−14	0.00793681	57.3781906
	rs7932326	0.0772058	0.0171159	7174	6.55748e−06	0.00282819	20.3413271
Phosphatidylcholine (18:2_18:2)	rs1077835	0.114534	0.019222	7174	2.6626e−09	0.00492455	35.4936465
	rs117017186	0.362427	0.0795703	7174	5.32894e−06	0.00288352	20.7404419
	rs117675848	0.320882	0.0654187	7174	9.53745e−07	0.0033425	24.0528089
	rs11869356	−0.0766319	0.0171208	7174	7.72162e−06	0.00278483	20.0285721
	rs13078306	−0.0818989	0.0176422	7174	3.50558e−06	0.00299493	21.5441599
	rs13150924	−0.111583	0.0250095	7174	8.25191e−06	0.00276708	19.9005444
	rs173539	0.0984345	0.0184699	7174	1.01409e−07	0.00394356	28.3951717
	rs174574	−0.22178	0.0168521	7174	4.16328e−39	0.02357301	173.147255
	rs56158036	0.295692	0.0637559	7174	3.57995e−06	0.00298935	21.5039032
	rs62238391	0.0863635	0.017876	7174	1.38372e−06	0.00324301	23.33451
	rs6498540	0.0934402	0.0177453	7174	1.43628e−07	0.00385003	27.7191389
	rs7717591	−0.095992	0.0178967	7174	8.4065e−08	0.00399415	28.7609398
	rs7798734	−0.081769	0.0176579	7174	3.70478e−06	0.00298017	21.4376993
	rs80315588	−0.0767847	0.0168318	7174	5.15184e−06	0.00289247	20.8049735
Sphingomyelin (d34:0)	rs1073042	−0.0823051	0.0182851	6207	6.87365e−06	0.00325358	20.2543754
	rs11631073	−0.0911807	0.0186999	6207	1.1084e−06	0.0038158	23.7677087
	rs1324162	0.0899835	0.0201129	6207	7.81063e−06	0.00321437	20.0095082
	rs16850360	0.227483	0.0504095	6207	6.51148e−06	0.00327016	20.3579084
	rs174535	−0.0976311	0.0183889	6207	1.13756e−07	0.0045208	28.1789563
	rs2808569	−0.0978734	0.0210823	6207	3.50945e−06	0.00346024	21.545343
	rs3026120	−0.207247	0.0341995	6207	1.43923e−09	0.00588157	36.7110691
	rs73015021	−0.15289	0.0315851	6207	1.32465e−06	0.00376076	23.4236025
	rs7814780	−0.0869509	0.0192999	6207	6.74575e−06	0.00325941	20.2907494
	rs8071514	0.0848261	0.0180916	6207	2.80367e−06	0.00352929	21.9768319
	rs934198	0.0978059	0.0198357	6207	8.3951e−07	0.00390171	24.3049698
Triacylglycerol (48:1)	rs10861498	−0.0952476	0.019381	7019	9.09524e−07	0.00342917	24.1452637
	rs12565526	0.0795461	0.0175846	7019	6.17408e−06	0.00290692	20.4573337
	rs1260326	−0.118511	0.0176221	7019	1.88871e−11	0.00640232	45.2145525
	rs147463852	−0.440217	0.096887	7019	5.61791e−06	0.00293259	20.6385336
	rs16970164	−0.202438	0.0425919	7019	2.04282e−06	0.00320818	22.5842817
	rs17192812	0.165787	0.0366097	7019	6.03367e−06	0.00291317	20.5014623
	rs3127050	0.0882816	0.018625	7019	2.17713e−06	0.00319069	22.4607401
	rs6079205	−0.0765162	0.0172598	7019	9.41841e−06	0.00279219	19.6476903
	rs6664147	−0.0967534	0.0216447	7019	7.93696e−06	0.0028387	19.9758636
	rs6804331	−0.0803188	0.0180013	7019	8.24665e−06	0.00282827	19.9022839
	rs7031238	0.0750969	0.0168493	7019	8.43547e−06	0.00282213	19.8589612
	rs7721676	−0.0907963	0.0204262	7019	8.91782e−06	0.00280715	19.7531962
	rs79152531	−0.188193	0.0360273	7019	1.80354e−07	0.00387243	27.2784527
Triacylglycerol (48:2)	rs10084264	0.172089	0.0363614	7071	2.25621e−06	0.0031577	22.3924789
	rs10861498	−0.0879739	0.0192951	7071	5.21205e−06	0.00293128	20.782164
	rs1260326	−0.137885	0.0175462	7071	4.45849e−15	0.00865786	61.7369316
	rs1832326	0.0832489	0.0187957	7071	9.59737e−06	0.00276666	19.6117859
	rs2546043	−0.0802241	0.0167299	7071	1.65631e−06	0.0032414	22.9879456
	rs3127050	0.0885169	0.0185552	7071	1.87213e−06	0.00320808	22.750883
	rs6664147	−0.0959268	0.0215573	7071	8.71861e−06	0.00279252	19.7955844
	rs6804331	−0.081343	0.0179395	7071	5.86932e−06	0.0028992	20.5540234
	rs72738698	0.101596	0.0220537	7071	4.15883e−06	0.00299232	21.2161921
	rs79152531	−0.194281	0.0359655	7071	6.80166e−08	0.00410978	29.1719594
Triacylglycerol (51:3)	rs10147474	−0.205161	0.0449078	7119	4.9914e−06	0.00292318	20.8652709
	rs1042034	0.107098	0.0186743	7119	1.01247e−08	0.00459889	32.8815311
	rs1047974	0.143421	0.0316816	7119	6.0771e−06	0.00287041	20.4875135
	rs111568723	0.242108	0.0546607	7119	9.58185e−06	0.00274823	19.6130834
	rs1260326	−0.13791	0.0174639	7119	3.27832e−15	0.00868365	62.3429025
	rs12635725	0.0753128	0.0167629	7119	7.13444e−06	0.00282742	20.1798184
	rs138427786	0.301941	0.0665321	7119	5.76122e−06	0.00288475	20.5901584
	rs139278484	0.252416	0.0563323	7119	7.53858e−06	0.0028124	20.0722828
	rs139500046	−0.37314	0.0798691	7119	3.03631e−06	0.00305659	21.8204652
	rs15285	−0.105751	0.0189806	7119	2.61547e−08	0.00434151	31.033236
	rs16996148	−0.148112	0.0334981	7119	9.93836e−06	0.00273861	19.5442098
	rs35332062	−0.150726	0.0254703	7119	3.41151e−09	0.00489506	35.0095235
	rs390082	0.150707	0.0313089	7119	1.51269e−06	0.00324415	23.1637678
	rs7846649	0.0948767	0.0212506	7119	8.1378e−06	0.00279218	19.9275873

**FIGURE 5:**
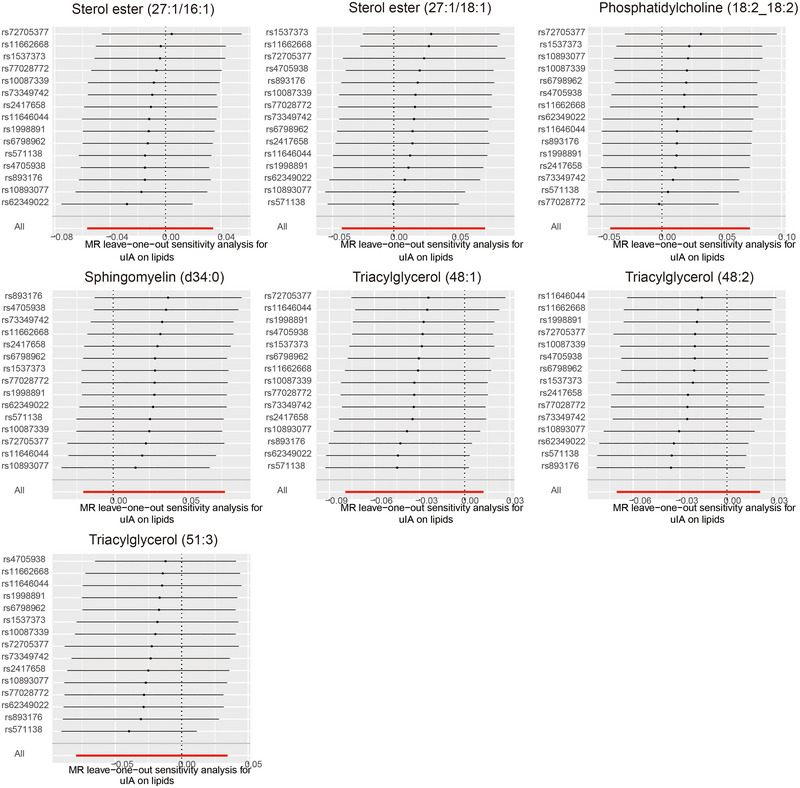
Leave‐one‐out plots for the reverse MR analyses.

In the reverse MR analysis, we used uIA GWAS data as exposure and the aforementioned seven lipids as outcomes, and detailed reverse MR results indicate no bidirectional causal relationship between these lipids and uIAs (Table 3). SNPs used as IVs are provided in Table 4. The sensitivity test results of this step are shown in Figure 5.

**TABLE 3:** Results of the reverse MR analyses.

Lipid	Method	nsnp	*β*	SE	*p* value	lo_ci	up_ci	OR	OR_lci95	OR_uci95	*p* value of Cochran's *Q* test	*p* value of plieotropy test
Sterol ester (27:1/16:1)	Inverse variance weighted	15	−0.0111488	0.02320979	0.63097773	−0.05664	0.03434233	0.98891307	0.94493416	1.03493884	0.476912826	0.467332845
	MR‐Egger	15	−0.0621994	0.07204397	0.40359054	−0.2034056	0.07900674	0.93969545	0.8159472	1.08221162	0.442002712	
	Weighted median	15	−0.0130895	0.03310102	0.69251794	−0.0779675	0.05178852	0.98699582	0.92499451	1.053153		
Sterol ester (27:1/18:1)	Inverse variance weighted	15	0.01605737	0.02979205	0.58989945	−0.0423351	0.0744498	1.01618699	0.95854856	1.07729126	0.058509544	0.937617013
	MR‐Egger	15	0.02328463	0.09570366	0.81157052	−0.1642945	0.2108638	1.02355784	0.84849208	1.23474417	0.040511803	
	Weighted median	15	−0.0073018	0.03345764	0.82724124	−0.0728788	0.05827513	0.99272476	0.92971351	1.0600066		
Phosphatidylcholine (18:2_18:2)	Inverse variance weighted	15	0.01513174	0.02977265	0.611283	−0.0432227	0.07348613	1.0152468	0.95769813	1.07625361	0.059329482	0.832808868
	MR‐Egger	15	0.03458989	0.09546036	0.72291435	−0.1525124	0.2216922	1.03519508	0.85854823	1.24818712	0.041962848	
	Weighted median	15	−0.0392082	0.03436573	0.25390795	−0.106565	0.02814868	0.96155053	0.89891662	1.02854859		
Sphingomyelin (d34:0)	Inverse variance weighted	15	0.02823919	0.02494714	0.25765083	−0.0206572	0.07713559	1.0286417	0.97955469	1.08018853	0.548985476	0.856623974
	MR‐Egger	15	0.04169092	0.07713488	0.59799539	−0.1094934	0.19287528	1.04257219	0.89628805	1.21273153	0.472648918	
	Weighted median	15	0.03776757	0.03525381	0.28403244	−0.0313299	0.10686504	1.03848983	0.9691558	1.11278406		
Triacylglycerol (48:1)	Inverse variance weighted	15	−0.0334031	0.02346	0.15449563	−0.0793847	0.01257854	0.96714866	0.92368455	1.01265799	0.462430251	0.353077668
	MR‐Egger	15	0.0327216	0.0725565	0.65943385	−0.1094891	0.17493233	1.03326283	0.8962919	1.1911656	0.455351514	
	Weighted median	15	−0.0477989	0.03367784	0.15581202	−0.1138075	0.01820966	0.95332548	0.89242976	1.01837647		
Triacylglycerol (48:2)	Inverse variance weighted	15	−0.0246066	0.02338199	0.29262771	−0.0704353	0.02122208	0.97569367	0.93198804	1.02144887	0.45914209	0.172845623
	MR‐Egger	15	0.07416298	0.0723576	0.32408123	−0.0676579	0.21598387	1.07698232	0.93458013	1.24108236	0.544650721	
	Weighted median	15	−0.0366348	0.03404725	0.28192728	−0.1033674	0.03009777	0.9640281	0.90179555	1.03055529		
Triacylglycerol (51:3)	Inverse variance weighted	15	−0.0225039	0.02910945	0.43947558	−0.0795584	0.03455061	0.97774742	0.92352407	1.03515442	0.080285299	0.085553138
	MR‐Egger	15	0.12382717	0.08309871	0.16005089	−0.0390463	0.28670065	1.13182024	0.96170617	1.33202541	0.185538013	
	Weighted median	15	−0.0105491	0.03405797	0.75675899	−0.0773027	0.05620452	0.98950634	0.9256096	1.05781401		

**TABLE 4:** SNPs used in the reverse MR analyses.

Lipid	SNP	*β*	SE	Sample size	*p* value	*R* ^2^	*F*
Sterol ester (27:1/16:1)	rs10087339	0.1966	0.0434	74022	6.005e−06	0.00027714	20.519928
	rs10893077	−0.2538	0.054	74022	2.634e−06	0.00029834	22.0894032
	rs11646044	−0.2051	0.0398	74022	2.534e−07	0.00035863	26.5554364
	rs11662668	−0.1761	0.0391	74022	6.554e−06	0.00027396	20.2839935
	rs1537373	−0.1954	0.0342	74022	1.075e−08	0.0004408	32.6426322
	rs1998891	−0.151	0.0339	74022	8.38e−06	0.00026796	19.8400501
	rs2417658	−0.2178	0.046	74022	2.23e−06	0.00030277	22.4175606
	rs4705938	0.1482	0.0335	74022	9.802e−06	0.00026432	19.5701908
	rs571138	−0.2042	0.0397	74022	2.75e−07	0.00035728	26.4556677
	rs62349022	−0.2468	0.0517	74022	1.839e−06	0.00030776	22.7875424
	rs6798962	−0.1876	0.0409	74022	4.612e−06	0.00028414	21.0381389
	rs72705377	−0.5121	0.1094	74022	2.857e−06	0.00029593	21.9110492
	rs73349742	0.8042	0.1775	74022	5.915e−06	0.00027724	20.5267262
	rs77028772	−0.2715	0.0567	74022	1.696e−06	0.00030966	22.92777
	rs893176	0.3235	0.0716	74022	6.158e−06	0.0002757	20.4131859
Sterol ester (27:1/18:1)	rs10087339	0.1966	0.0434	74022	6.005e−06	0.00027714	20.519928
	rs10893077	−0.2538	0.054	74022	2.634e−06	0.00029834	22.0894032
	rs11646044	−0.2051	0.0398	74022	2.534e−07	0.00035863	26.5554364
	rs11662668	−0.1761	0.0391	74022	6.554e−06	0.00027396	20.2839935
	rs1537373	−0.1954	0.0342	74022	1.075e−08	0.0004408	32.6426322
	rs1998891	−0.151	0.0339	74022	8.38e−06	0.00026796	19.8400501
	rs2417658	−0.2178	0.046	74022	2.23e−06	0.00030277	22.4175606
	rs4705938	0.1482	0.0335	74022	9.802e−06	0.00026432	19.5701908
	rs571138	−0.2042	0.0397	74022	2.75e−07	0.00035728	26.4556677
	rs62349022	−0.2468	0.0517	74022	1.839e−06	0.00030776	22.7875424
	rs6798962	−0.1876	0.0409	74022	4.612e−06	0.00028414	21.0381389
	rs72705377	−0.5121	0.1094	74022	2.857e−06	0.00029593	21.9110492
	rs73349742	0.8042	0.1775	74022	5.915e−06	0.00027724	20.5267262
	rs77028772	−0.2715	0.0567	74022	1.696e−06	0.00030966	22.92777
	rs893176	0.3235	0.0716	74022	6.158e−06	0.0002757	20.4131859
Phosphatidylcholine (18:2_18:2)	rs10087339	0.1966	0.0434	74022	6.005e−06	0.00027714	20.519928
	rs10893077	−0.2538	0.054	74022	2.634e−06	0.00029834	22.0894032
	rs11646044	−0.2051	0.0398	74022	2.534e−07	0.00035863	26.5554364
	rs11662668	−0.1761	0.0391	74022	6.554e−06	0.00027396	20.2839935
	rs1537373	−0.1954	0.0342	74022	1.075e−08	0.0004408	32.6426322
	rs1998891	−0.151	0.0339	74022	8.38e−06	0.00026796	19.8400501
	rs2417658	−0.2178	0.046	74022	2.23e−06	0.00030277	22.4175606
	rs4705938	0.1482	0.0335	74022	9.802e−06	0.00026432	19.5701908
	rs571138	−0.2042	0.0397	74022	2.75e−07	0.00035728	26.4556677
	rs62349022	−0.2468	0.0517	74022	1.839e−06	0.00030776	22.7875424
	rs6798962	−0.1876	0.0409	74022	4.612e−06	0.00028414	21.0381389
	rs72705377	−0.5121	0.1094	74022	2.857e−06	0.00029593	21.9110492
	rs73349742	0.8042	0.1775	74022	5.915e−06	0.00027724	20.5267262
	rs77028772	−0.2715	0.0567	74022	1.696e−06	0.00030966	22.92777
	rs893176	0.3235	0.0716	74022	6.158e−06	0.0002757	20.4131859
Sphingomyelin (d34:0)	rs10087339	0.1966	0.0434	74022	6.005e−06	0.00027714	20.519928
	rs10893077	−0.2538	0.054	74022	2.634e−06	0.00029834	22.0894032
	rs11646044	−0.2051	0.0398	74022	2.534e−07	0.00035863	26.5554364
	rs11662668	−0.1761	0.0391	74022	6.554e−06	0.00027396	20.2839935
	rs1537373	−0.1954	0.0342	74022	1.075e−08	0.0004408	32.6426322
	rs1998891	−0.151	0.0339	74022	8.38e−06	0.00026796	19.8400501
	rs2417658	−0.2178	0.046	74022	2.23e−06	0.00030277	22.4175606
	rs4705938	0.1482	0.0335	74022	9.802e−06	0.00026432	19.5701908
	rs571138	−0.2042	0.0397	74022	2.75e−07	0.00035728	26.4556677
	rs62349022	−0.2468	0.0517	74022	1.839e−06	0.00030776	22.7875424
	rs6798962	−0.1876	0.0409	74022	4.612e−06	0.00028414	21.0381389
	rs72705377	−0.5121	0.1094	74022	2.857e−06	0.00029593	21.9110492
	rs73349742	0.8042	0.1775	74022	5.915e−06	0.00027724	20.5267262
	rs77028772	−0.2715	0.0567	74022	1.696e−06	0.00030966	22.92777
	rs893176	0.3235	0.0716	74022	6.158e−06	0.0002757	20.4131859
Triacylglycerol (48:1)	rs10087339	0.1966	0.0434	74022	6.005e−06	0.00027714	20.519928
	rs10893077	−0.2538	0.054	74022	2.634e−06	0.00029834	22.0894032
	rs11646044	−0.2051	0.0398	74022	2.534e−07	0.00035863	26.5554364
	rs11662668	−0.1761	0.0391	74022	6.554e−06	0.00027396	20.2839935
	rs1537373	−0.1954	0.0342	74022	1.075e−08	0.0004408	32.6426322
	rs1998891	−0.151	0.0339	74022	8.38e−06	0.00026796	19.8400501
	rs2417658	−0.2178	0.046	74022	2.23e−06	0.00030277	22.4175606
	rs4705938	0.1482	0.0335	74022	9.802e−06	0.00026432	19.5701908
	rs571138	−0.2042	0.0397	74022	2.75e−07	0.00035728	26.4556677
	rs62349022	−0.2468	0.0517	74022	1.839e−06	0.00030776	22.7875424
	rs6798962	−0.1876	0.0409	74022	4.612e−06	0.00028414	21.0381389
	rs72705377	−0.5121	0.1094	74022	2.857e−06	0.00029593	21.9110492
	rs73349742	0.8042	0.1775	74022	5.915e−06	0.00027724	20.5267262
	rs77028772	−0.2715	0.0567	74022	1.696e−06	0.00030966	22.92777
	rs893176	0.3235	0.0716	74022	6.158e−06	0.0002757	20.4131859
Triacylglycerol (48:2)	rs10087339	0.1966	0.0434	74022	6.005e−06	0.00027714	20.519928
	rs10893077	−0.2538	0.054	74022	2.634e−06	0.00029834	22.0894032
	rs11646044	−0.2051	0.0398	74022	2.534e−07	0.00035863	26.5554364
	rs11662668	−0.1761	0.0391	74022	6.554e−06	0.00027396	20.2839935
	rs1537373	−0.1954	0.0342	74022	1.075e−08	0.0004408	32.6426322
	rs1998891	−0.151	0.0339	74022	8.38e−06	0.00026796	19.8400501
	rs2417658	−0.2178	0.046	74022	2.23e−06	0.00030277	22.4175606
	rs4705938	0.1482	0.0335	74022	9.802e−06	0.00026432	19.5701908
	rs571138	−0.2042	0.0397	74022	2.75e−07	0.00035728	26.4556677
	rs62349022	−0.2468	0.0517	74022	1.839e−06	0.00030776	22.7875424
	rs6798962	−0.1876	0.0409	74022	4.612e−06	0.00028414	21.0381389
	rs72705377	−0.5121	0.1094	74022	2.857e−06	0.00029593	21.9110492
	rs73349742	0.8042	0.1775	74022	5.915e−06	0.00027724	20.5267262
	rs77028772	−0.2715	0.0567	74022	1.696e−06	0.00030966	22.92777
	rs893176	0.3235	0.0716	74022	6.158e−06	0.0002757	20.4131859
Triacylglycerol (51:3)	rs10087339	0.1966	0.0434	74022	6.005e−06	0.00027714	20.519928
	rs10893077	−0.2538	0.054	74022	2.634e−06	0.00029834	22.0894032
	rs11646044	−0.2051	0.0398	74022	2.534e−07	0.00035863	26.5554364
	rs11662668	−0.1761	0.0391	74022	6.554e−06	0.00027396	20.2839935
	rs1537373	−0.1954	0.0342	74022	1.075e−08	0.0004408	32.6426322
	rs1998891	−0.151	0.0339	74022	8.38e−06	0.00026796	19.8400501
	rs2417658	−0.2178	0.046	74022	2.23e−06	0.00030277	22.4175606
	rs4705938	0.1482	0.0335	74022	9.802e−06	0.00026432	19.5701908
	rs571138	−0.2042	0.0397	74022	2.75e−07	0.00035728	26.4556677
	rs62349022	−0.2468	0.0517	74022	1.839e−06	0.00030776	22.7875424
	rs6798962	−0.1876	0.0409	74022	4.612e−06	0.00028414	21.0381389
	rs72705377	−0.5121	0.1094	74022	2.857e−06	0.00029593	21.9110492
	rs73349742	0.8042	0.1775	74022	5.915e−06	0.00027724	20.5267262
	rs77028772	−0.2715	0.0567	74022	1.696e−06	0.00030966	22.92777
	rs893176	0.3235	0.0716	74022	6.158e−06	0.0002757	20.4131859

## 4 Discussion

IA is a common cerebrovascular disorder with diverse clinical manifestations, ranging from asymptomatic cases to those causing neurological dysfunction or compression of adjacent structures (W. Li et al. 2021). However, the most severe consequence is the rupture of IAs, leading to SAH, which poses a life‐threatening condition. Therefore, investigating the risk factors for the formation of IAs is of paramount importance.

Recent research has revealed the critical role of lipid metabolism in the onset of IAs. For instance, an MR study found that genetically determined levels of HDL‐C and LDL‐C were associated with a reduced risk of IAs and IA rupture, shedding light on the impact of lipid‐modifying drugs on IAs (Karhunen et al. 2021). However, these findings were not widely accepted, for another study did not identify a correlation between IA and TG or LDL‐C (Zhang et al. 2022).

Furthermore, certain proteins and genetic variations related to lipid metabolism have been confirmed to be associated with IAs. For instance, apolipoprotein E (APOE), a key regulatory factor in lipid metabolism, has been linked to genetic susceptibility to arterial aneurysms (Liu et al. 2016). Differences in gene expression related to lipid metabolism in IA patients also underscore the potential role of lipid metabolism in the pathogenesis of IAs, such as increased expression of the LDLR gene in IAs and specific genotypes (A/G) and alleles (A) of the APOA1 gene contributing to an increased risk of IAs (Synowiec et al. 2016).

In terms of mechanisms, pathological changes in IAs involve lipid deposition and alterations in the vascular wall structure and inflammatory responses within the damaged endothelial layer, induced by the interplay of lipid metabolism and blood flow. This ultimately leads to apoptosis of endothelial cells and smooth muscle cells, weakening the mechanical strength of the vascular wall, causing local outward bulging, and resulting in the formation of an arterial aneurysm (J. Jin et al. 2022).

However, former studies have focused solely on standard lipids such as HDL‐C, LDL‐C, TG, and TC. This makes our research strengths stand out. Firstly, we utilized data from a GWAS research that encompasses 179 lipid variants, enabling us to explore the impact of these diverse lipid isomers on the formation of uIAs and more accurately predict the risk of uIAs. (Ottensmann et al. 2023)

The obtained results highlight the significant impact of these lipid isomers on IAs, which has been scarcely explored in previous research, particularly in relation to vascular‐related disorders. The novelty of this study is underscored by the limited recognition of these isomers in prior investigations. Although the direct traces of these isomers were absent in previous studies, we have nonetheless summarized the roles of the major lipid classes they belong to.

For SEs, studies have indicated that their elevation may contribute to the process of “lipid raft aging,” leading to increased viscosity and reduced fluidity of lipid rafts (Diaz et al. 2023), and may be advantageous for the pro‐inflammatory state in cerebral tissue (Stables and Gilroy 2011). As IA is a cerebrovascular disease influenced by hemodynamics, we sought relevant research on the impact of SEs on hemodynamics and found only a mild effect (Hallikainen et al. 2006). Interestingly, our research identified two SE isomers with opposing effects on IAs, suggesting a potential mutual offsetting of the isomers leading to an overall diminished effect of SEs.

PCs are major components of mitochondrial membranes and are crucial for the synthesis of mature phospholipids in the heart, playing a vital role in maintaining mitochondrial function (X. Li et al. 2015). Limited information is available on the specific impact of PC (18:2_18:2) isomer on cerebrovascular diseases, as only one previous study associated this subtype with the severity of bronchiolitis (Kyo et al. 2023). On the contrary, other PC subtypes such as PC (22:6/18:2), PC (22:6/18:1), PC (20:4/16:1), and PC (16:1/18:3) have shown potential implications in diseases like myocardial infarction, and PC (16:0/16:0) has been associated with hypertension (Dong et al. 2017, Shoghli et al. 2023). Despite this, it is noteworthy that the PC (18:2_18:2) constitutes a significant proportion (34%) of soybean PC, suggesting a potential association between soybean intake and IA risk, although further research is needed (Le Grandois et al. 2009).

TAG, also known as TG, is acknowledged as a crucial risk factor in the formation of IAs, playing a key role in atherosclerosis‐related CVDs. Intracranial atherosclerosis leads to the deposition of lipid plaques, endothelial cell damage, and rupture of elastic fibers, resulting in weakened walls of intracranial arteries. Elevated intracranial pressure can cause localized dilation of these weakened arteries, ultimately leading to the formation of intracranial (Gutierrez et al. 2022; Holmstedt et al. 2013). However, recent MR studies investigating the relationship between lipids and IAs have not found any association between TGs and IAs (Karhunen et al. 2021; Zhang et al. 2022). Our study identified a specific association with only the following TAG subspecies: TAG (48:1), TAG (48:2), and TAG (51:3). This finding may offer new directions for understanding the role of TAGs in the context of IAs.

SM belongs to the class of sphingolipids and serves as a component of cell membranes, functioning as a bioactive signaling molecule (Ruangsiriluk et al. 2012). Clinically, plasma levels of SM have been associated with the progression of CADs and are considered an independent risk factor for CADs (Jiang et al. 2000). Unfortunately, there is a lack of research specifically addressing this particular isomer in the context of IAs.

In this study, we employed MR methodology, enhancing our ability to formulate causal hypotheses and increasing confidence in our findings. This approach mitigates various issues in observational studies, including confounding, selection bias, and memory bias, thereby aiding in clarifying relationships between variables and providing more reliable causal inferences (Smith and Ebrahim 2003). Additionally, the use of a two‐sample MR design allows for the combination of nonoverlapping exposure and outcome data to reduce bias.

There are still some limitations to our study: Firstly, due to the limited number of SNPs filtered by the conventional GWAs significance threshold (*p* < 5e−8), we lowered the standard to *p* < 1e−5, but rigorous pleiotropy and sensitivity tests helped remedy this deficiency. Secondly, MR analysis is influenced by demographic factors and genetic sequencing errors, and the current study population consists of individuals of European descent, limiting its generalizability. Furthermore, while MR analysis can provide evidence of causality, the interpretation of biological mechanisms may still be complex and require further experimental research. At last, we only focused on the formation of IAs and lacked a study on SAH. Further studies are needed to investigate whether these lipids have an effect on the rupture of IA.

## 5 Conclusion

Through MR analysis, we identified seven lipids that have a causal relationship with IAs, potentially offering new perspectives and directions for the risk assessment of IAs.

## Author Contributions

**Junqing Yan**: conceptualization, methodology, software, data curation, supervision, formal analysis, validation, visualization, writing–review and editing, writing – original draft, investigation, resources, project administration.

## Conflicts of Interest

The author declares no conflicts of interest.

### Peer Review

The peer review history for this article is available at https://publons.com/publon/10.1002/brb3.70435

## Declaration

The work has not been fully or partially published previously and is not under consideration for publication elsewhere.

Its publication is approved by all authors, and if accepted, it will not be published elsewhere in the same form, in English or in any other language, including electronically, without the written consent of the copyright holder.

## Data Availability

The data that supports the findings of this study are available from the corresponding author upon reasonable request.
